# Capsulotomy opening diameter outcomes in aphakic eyes after primary congenital cataract removal and its association

**DOI:** 10.3389/fped.2023.1062144

**Published:** 2023-02-21

**Authors:** Qihui Zhao, Pingjun Chang, Yinying Zhao, Dandan Wang, Yune Zhao

**Affiliations:** ^1^ Eye Hospital and School of Ophthalmology and Optometry, Wenzhou Medical University, Wenzhou, China; ^2^ National Clinical Research Center for Ocular Diseases, Wenzhou, China; ^3^ Ningbo First Hospital, Ningbo, China

**Keywords:** change, congenital cataract, capsulotomy opening diameter, association, aphakic eye

## Abstract

**Aim:**

To observe the change of capsulotomy opening diameter (COD) in aphakic eyes after primary congenital cataract removal and investigate its influencing factors.

**Methods:**

Ocular parameters, including corneal diameter (CD), axial length (AL), anterior and posterior COD (ACOD, PCOD), and age at surgery were recorded at primary congenital cataract removal and secondary intraocular lens implantation. The concentrations of 15 kinds of cytokines in aqueous humor samples collected at the primary surgery were detected. The change (Δ) of COD between two surgeries were described, and its association was analyzed.

**Results:**

Fifty eyes from 33 patients with congenital cataract who underwent primary and secondary surgery were enrolled. The changes in ACOD and PCOD were not statistically significant on the whole. ΔACOD was positively correlated with ΔCD and the concentrations of PDGF-AA, VEGF and TGF-β1. The concentration of FGF-2 and the interval between two surgeries showed negative correlations with ΔACOD and ΔPCOD.

**Conclusion:**

COD in aphakic eyes kept changing after primary surgery. The positive correlation between ΔACOD and ΔCD manifested the enlargement of ACOD was influenced by lateral eye growth. Meanwhile, ΔACOD was also associated with cytokines, indicating postoperative inflammation promoted the ACOD constriction.

## Introduction

Congenital cataract, with an overall prevalence of 0.63–9.7 per 10,000 people, is the main cause of childhood blindness worldwide (1, 2). As a precondition for visual rehabilitation, surgery is now a safe and effective intervention for infant patients (3). Due to their undeveloped anatomical structure, majority of children <2-year-old routinely undergo secondary intraocular lens (IOL) implantation at an older age (4).

Sulcus and capsular bag are the most common positions to house an IOL during secondary surgery. The procedure of sulcus IOL implantation is relatively easier, but with higher rates of postoperative complications, such as pigment dispersion, elevated intraocular pressure and IOL decentration (5, 6). In-the-bag IOL implantation, with the advantage of conforming to physiological structure, better IOL centration and lower rates of postoperative complications, may be a better long-term choice for patients (6, 7).

Capsulotomy opening diameter (COD) is a key factor in IOL stability. Published studies had recommended an anterior COD (ACOD) of 4.0–5.0 mm and a posterior COD (PCOD) of 3.0–4.0 mm for optimal capsular outcome for secondary IOL implantation (8, 9). Along with the eye ball growth and inflammatory response, COD seemed to go through uncertain changes postoperatively such as capsulotomy opening area opacity and capsular constriction (10). Cases whose ACOD and PCOD changed similarly or inversely were occasionally seen, some of which even lost the chance to undergo in-the-bad IOL implantation due to severe COD change. Thus, it was necessary to figure out the change of COD. However, previously studies rarely focused on COD itself.

This study aimed to observe the change of COD in aphakic eyes after primary congenital cataract removal, and to investigate its influencing factors, in order to discover the change rule of COD.

## Methods

### Patients and enrolment criteria

Patients who were diagnosed with congenital cataract by experienced ophthalmologists and underwent primary cataract removal and secondary IOL implantation at the Eye Hospital of Wenzhou Medical University (Hangzhou branch) were preliminarily enrolled. Complete follow-up data and clear operation videos that showed COD were requisite. Exclusion criteria included ophthalmic diseases (microcornea, microphthalmia, posterior capsule defect and persistent fetal vasculature), and systemic disorders (Marfan syndrome). Patients with severe capsular fibrosis, who needed scissors or a vitrector to remove part of capsule, and whose pupils could not be dilated during surgery, were also excluded.

This study was conducted following the tenets of the Declaration of Helsinki, adhered to the ARVO statement on human subjects. It was approved by the Ethics Review Committee of Wenzhou Medical University. Informed consent forms were obtained from the legal guardians of the patients.

### Surgical technique

All surgeries were performed by the same experienced pediatric cataract surgeon (Yune Zhao) under general anesthesia using a 23-gauge microincision vitrectomy system (Accurus, Alcon Laboratories, Fort Worth, TX, United States).

Primary surgery included lensectomy, posterior capsulotomy and limited anterior vitrectomy. Two 1.0-mm clear corneal paracenteses were created at 3 and 9 o'clock using a diamond knife. A 23-gauge irrigating cannula was inserted *via* one port to maintain the anterior chamber, while the other 23-gauge vitrector was inserted *via* the other port to create a stab opening in the anterior capsule. The vitrector hole was then extended until an anterior capsulotomy opening of approximately 4.0–5.0 mm diameter was created. The cortex was aspirated in irrigation/aspiration mode. A posterior capsulotomy opening of approximately 3.0–4.0 mm diameter was performed with the vitrector. About one third of the anterior vitreous volume was removed using the same vitrectomy setting.

Compared to primary surgery, an additional scleral tunnel at 12 o'clock was made for IOL insertion at secondary surgery. After reopening the residual capsule with an iris spatula or capsulorhexis forceps and aspirating the cortex in the peripheral bag with a vitrector, the viscoelastic material was filled into the rebuilt capsular bag and a suitable IOL was placed in the bag.

### Ocular parameters

The age at surgery was recorded. Axial length (AL) was measured *via* an A-scan (Axis nano, Quantel Medical, Cournon, France) under sedation before surgery. Corneal diameter (CD) was measured after general anethesia at the beginning of surgery using calipers from the nasal to the temporal inner side of the limbus transition zone.

**Figure 1:**
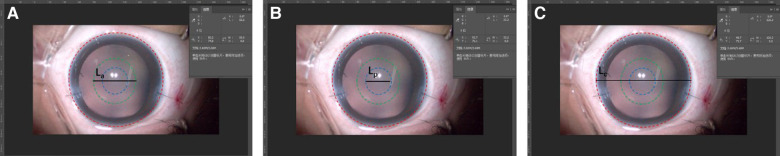
Measurements of anterior capsulotomy opening diameter (ACOD) and posterior capsulotomy opening diameter (PCOD) using adobe photoshop CC 2018. The red, green, and blue circles represented the cornea and, anterior and posterior capsulorhexis opening, while La, Lp, and Lc represent ACOD, PCOD and corneal diameter (CD), respectively, in units of pixels. As the value of CD (mm) was measured, the values of ACOD and PCOD (mm) could be calculated by equal-scale conversion: ACOD=CD×La/Lc,PCOD=CD×Lp/Lc.

The measurements of ACOD and posterior OCD (PCOD) were performed using Adobe Photoshop CC 2018 (Adobe Systems Inc., CA, United States) (Figure 1). First, we captured a legible image that clearly showed ACOD and PCOD from the operation video. In the video of the primary surgery, an image was captured under a stable anterior chamber after suturing two corneal incisions. As for the secondary surgery, images were captured after separating the fused anterior and posterior capsule and when the anterior chamber was well maintained by a 23-gauge infusion cannula with the pressure of 30 cmH_2_O. Second, we measured the length of CD, ACOD and PCOD from the nasal edge to the temporal edge horizontally *via* Adobe Photoshop CC 2018 in units of pixels. Finally, since the value of CD in units of millimeter had already been measured before, the values of ACOD and PCOD in units of millimeter were calculated by equal-scale conversion.

**Figure 2:**
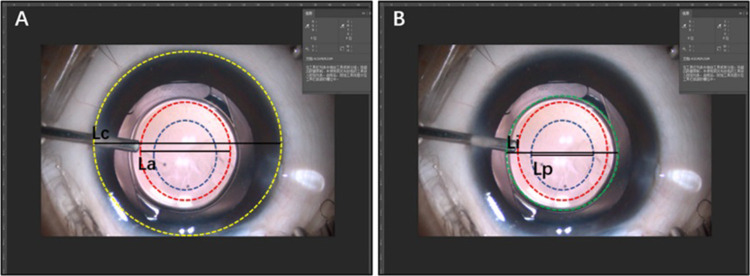
Verification of the accuracy of the measurement method of capsulotomy opening diameter (COD). (**A**) The method converted the values of ACOD and PCOD (mm) through the value of CD (mm). (**B**) The method converted the values of ACOD and PCOD (mm) through the value of the optical surface diameter of the intraocular lens (6 mm).

As all parameters used for the equal-scale conversion were measured manually, we used a fixed optical surface diameter of IOL (6 mm) to test the accuracy of this method (Figure 2). First, we captured a legible image that clearly showed ACOD and PCOD from the operation video of primary IOL implantation. Second, we measured the length of CD, ACOD, PCOD and IOL in units of pixels from the nasal edge to the temporal edge horizontally using Adobe Photoshop CC 2018. Third, as the value of CD (mm) had already been measured before and the optical surface diameter of IOL was fixed, the values of ACOD and PCOD (mm) could be calculated by equal-scale conversion through CD and IOL. Finally, the values of ACOD and PCOD calculated using CD and IOL were compared.

The change (Δ) of the parameters between two surgeries was defined as the parameter of secondary surgery minus that of primary surgery.

### Collection and detection of aqueous humor samples

Aqueous humor samples were collected at the beginning of primary surgery using a 29-gauge needle *via* limbal paracentesis. All samples were collected before any intraocular operation to avoid the influence of surgical stimulation. Considering that the anterior chambers in infant patients were narrow, only 100 μl of aqueous humor was collected. After being transferred to aseptic EP tubes marked with the information of the patients, the samples were immediately stored in −80°C refrigerator until analysis.

By referring to the published studies focusing on the association between cytokines and capsular structure, a total of 15 kinds of cytokines were selected for detection, including IL-6, IL-10, IL-15, TNF-α, IFN-γ, MCP-1, IP-10, G-CSF, FGF-2, PDGF-AA, VEGF, EGF, TGF-β1, TGF-β2, and TGF-β3. The concentrations of these cytokines were measured using Luminex xMAP technology with multi-analyte profiling beads (Lincoplex cytokine/chemokine multiplex kit, HCYTO-60K; Millipore Corporation, Billerica, MA).

### Statistical analysis

Data were analyzed using SPSS 22.0 (SPSS, Armonk, NY, United States). Normally distributed data were expressed as mean ± standard deviation. Two-tailed paired t test was used to compare the means between the primary and secondary parameters. The correlations between normally distributed parameters were analyzed using Pearson's correlation analysis. For data that did not fit the normal distribution, values were recorded as median and 25th to 75th interquartile range. Wilcoxon signed-rank test was used to compare the primary and secondary parameters. Spearman correlation analysis was used to analyze the correlations. A two-tailed *P* value less than 0.05 was considered statistically significant for all tests.

## Results

### Baseline data

**Figure 3:**
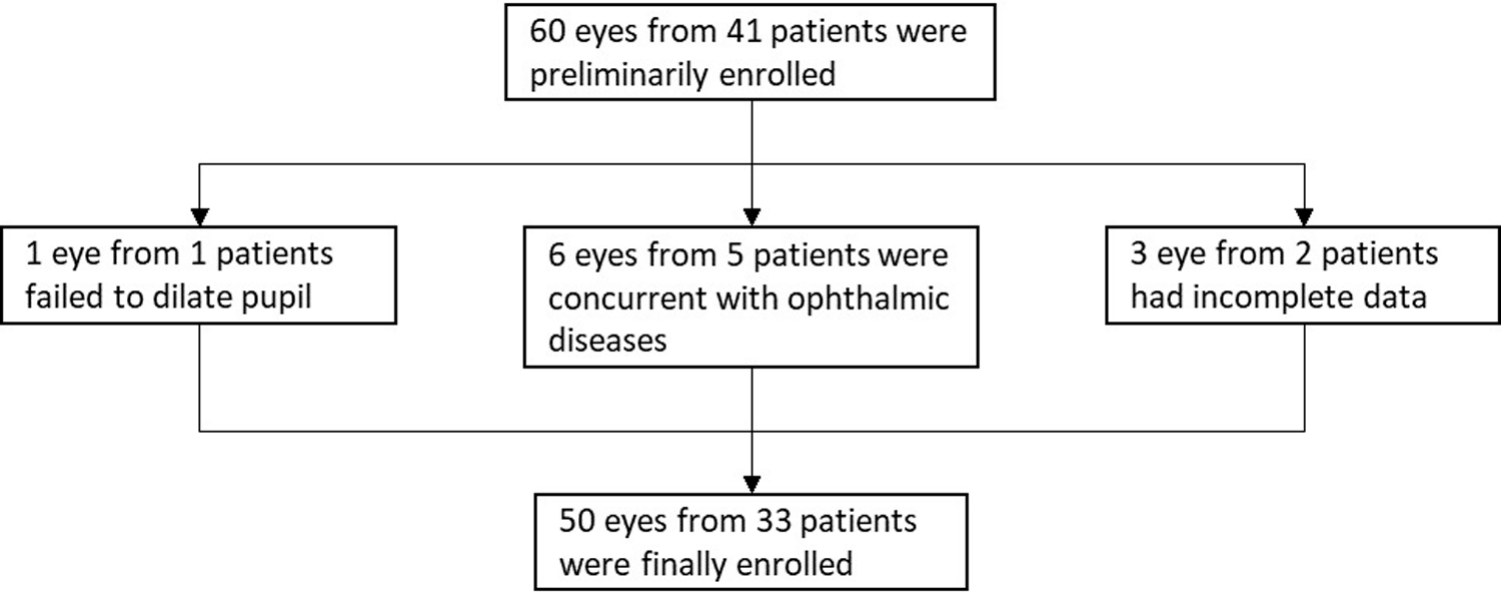
Detailed change in capsulotomy opening diameter (COD) between the two surgeries.

Sixty eyes from 41 congenital cataract patients were preliminarily enrolled. One eye from 1 patient, whose pupil failed to be dilated, was excluded. Six eyes from 5 patients were excluded due to concurrent ophthalmic diseases. Three eyes from 2 patients were excluded for incomplete data. Finally, 50 eyes from 33 patients who underwent primary and secondary surgery between February 2016 and April 2020 were enrolled (Figure 3). Demographic data for the enrolled patients are summarized in Table 1.

**Table 1:** Baseline data of the enrolled patients.

Characteristics	Number (%)
Sex (*n* = 33)	
Male	18 (54.5%)
Female	15 (45.5%)
Cataract laterality (*n* = 33)	
Bilateral	17 (51.5%)
Unilateral	16 (48.5%)
Eyes (*n* = 50)	
Right	24 (48.0%)
Left	26 (52.0%)
Age at primary surgery (months)	4.28 ± 1.64
Age at secondary surgery (months)	29.30 ± 7.44

**Table 2:** Intraclass correlation coefficient between the values of ACOD and PCOD converted by CD and IOL.

Conversion method	CD	IOL	ICC	*P*
ACOD (mm)	5.32 ± 0.19	5.27 ± 0.17	0.890	0.000
PCOD (mm)	4.29 ± 0.24	4.20 ± 0.20	0.856	0.000

ICC, intraclass correlation coefficient.

### Accuracy verification of the measurement method of COD

Twenty eyes that underwent primary IOL implantation were selected to test the accuracy of the measurement method. The values of ACOD and PCOD converted by CD and IOL were compared in Table 2. The result indicated that our method to measure COD in aphakic eyes was reliable.

### Change of COD between primary and secondary surgery

The values of the ocular parameters were presented as mean ± standard deviation and were summarized in Table 3. No significant difference in ACOD and PCOD between two surgeries was found.

**Table 3:** Mean ± standard deviation of ocular parameters collected at the primary and secondary surgery.

	Primary surgery	Secondary surgery	Change	*P* value
AL (mm)	18.72 ± 0.94	21.12 ± 1.54	2.41 ± 1.23	≤0.001
CD (mm)	9.40 ± 0.53	10.05 ± 0.66	0.65 ± 0.41	≤0.001
Age (months)	4.28 ± 1.64	29.30 ± 7.44	25.02 ± 7.24	≤0.001
ACOD (mm)	5.18 ± 0.42	5.13 ± 0.77	−0.05 ± 0.77	0.636
PCOD (mm)	3.82 ± 0.55	4.00 ± 0.81	0.17 ± 0.85	0.152

AL, axial length; CD, corneal diameter; ACOD, anterior capsulotomy opening diameter; PCOD, posterior capsulotomy opening diameter.

### Correlation of ΔCOD with ocular parameters and baseline data

**Figure 4:**
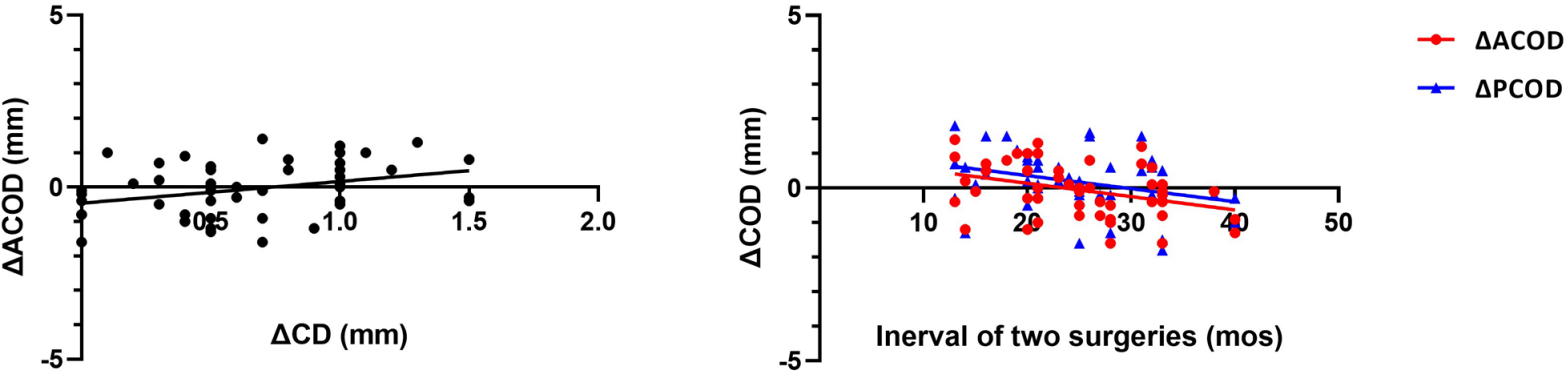
Comparisons of the concentrations of IL-15, TNF-α, MCP-1, G-CSF, and FGF-2 in the aqueous humor samples collected at the primary and secondary surgery. PAQ, aqueous humor sample collected at primary surgery; SAQ, aqueous humor sample collected at secondary surgery.

ΔACOD showed a positive correlation with ΔCD (*r*^2^ = 0.113, *y* = 0.63*x + 0.46, *P* = 0.017) (Figure 4), but no similar correlation was observed between ΔPCOD and ΔCD. Negative correlations were shown between the interval of two surgeries with ΔACOD (*r*^2^ = 0.131, *y* = −0.04*x + 0.91, *P* = 0.010) and ΔPCOD (*r*^2^ = 0.104, *y* = −0.04*x + 1.12, *P* = 0.022) (Figure 4). No significant correlation was observed between ΔCOD and ΔAL, age at surgery, sex and eye type.

### Correlation of ΔCOD with the concentrations of cytokines

A total of 50 samples from 50 eyes of 33 patients were collected. The concentrations of cytokines in the aqueous humor samples are presented as the median and 25th to 75th interquartile range (Table 4).

**Table 4:** Median and 25th to 75th interquartile range of the concentrations of cytokines in the aqueous humor samples collected at the primary surgery.

Cytokines	Concentration (pg/ml)
IL-6	6.75 (4.07, 24.65)
IL-10	45.49 (1.03, 141.73)
IL-15	3.43 (1.49, 7.33)
TNF-α	2.36 (0.64, 3.59)
IFN-γ	40.62 (36.15, 43.39)
MCP-1	1024.50 (675.61, 2026.79)
IP-10	46.55 (24.18, 106.57)
G-CSF	10.76 (5.39, 13.04)
FGF-2	10.44 (0.00, 16.24)
PDGF-AA	363.23 (285.45, 526.98)
VEGF	123.58 (77.68, 240.12)
EGF	13.00 (12.33, 16.53)
TGF-β1	7.83 (0.00, 34.21)
TGF-β2	2227.22 (1025.73, 4175.93)
TGF-β3	19.73 (14.25, 54.09)

**Figure 5:**
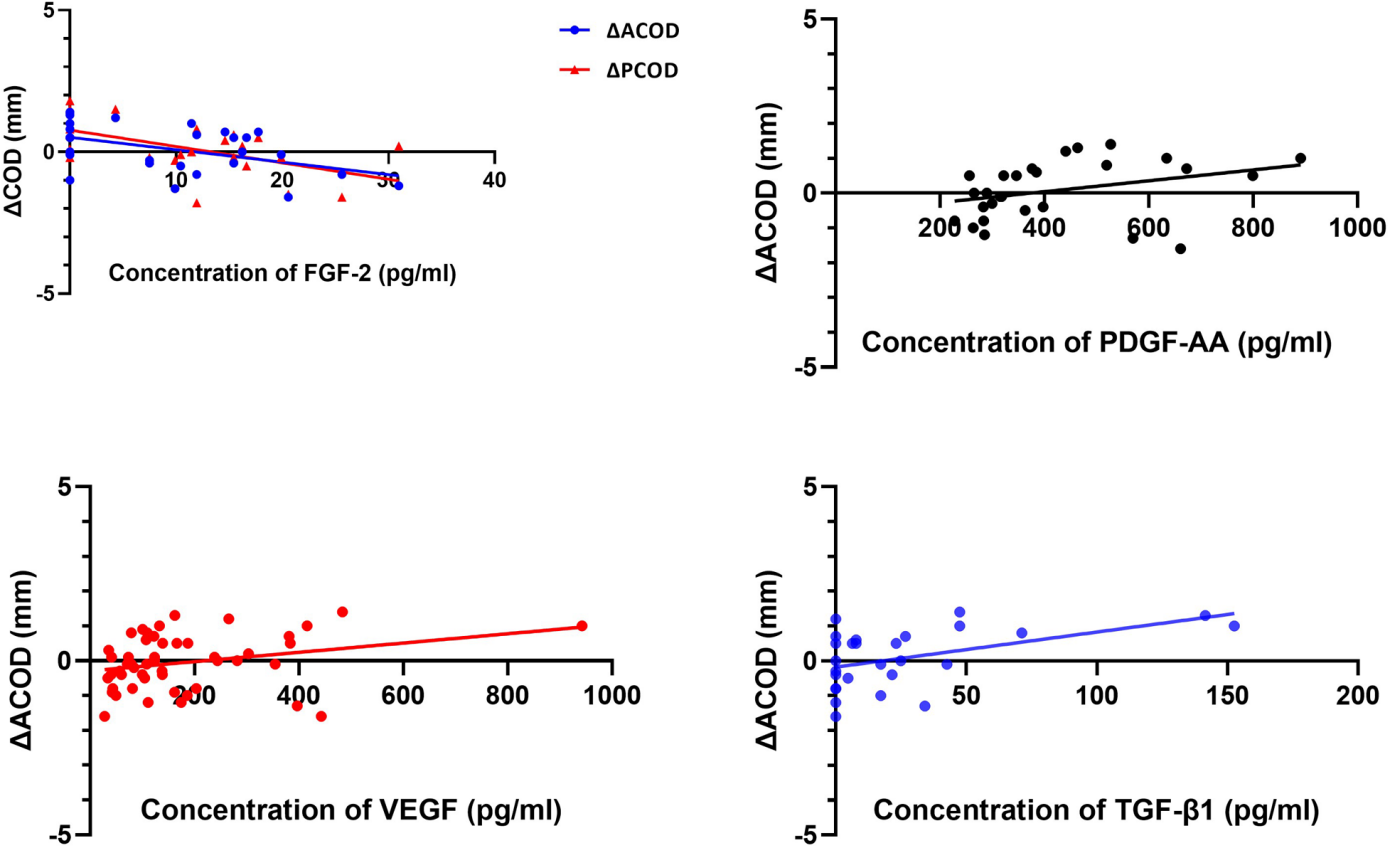
Correlation between ΔCOD and the concentrations of FGF-2, PDGF-AA, VEGF and TGF-β1.

Negative correlations were observed between the concentrations of FGF-2 with ΔACOD (*r*^2^ = 0.216, *y* = −0.04*x + 0.52, *P* = 0.037) and ΔPCOD (*r*^2^ = 0.319, *y* = −0.06*x + 0.77, *P* = 0.003) (Figure 5). Positive correlations were observed between ΔACOD with the concentrations of PDGF-AA (*r*^2^ = 0.108, *y* = 1.56 × 10^−3^*x − 0.58, *P* = 0.016), VEGF (*r*^2^ = 0.080, *y* = 1.33 × 10^−3^*x − 0.29, *P* = 0.033), and TGF-β1 (*r*^2^ = 0.225, *y* = 0.001*x − 0.18, *P* = 0.013) (Figure 5).

## Discussion

In consideration of the postoperative complications and the complexity of IOL power calculation, for infants younger than 2 years old, especially under 6 months, lens removal with posterior capsulotomy and limited anterior vitrectomy was the more common surgery choice (4, 11). At secondary IOL implantation, only those whose residual peripheral capsule was intact with suitable COD could accept in-the-bag IOL implantation.

In a previous study, COD was measured with a self-made measuring needle with a laser marker (1 mm scale) (12). This measurement method ignored the indication error caused by the angel between the capsulotomy opening plane and the measuring needle. In this study, COD was actually calculated by equal-scale conversion. Besides, we verified the accuracy of this method with a fixed optical surface diameter of IOL (6 mm).

In the enrolled aphakic eyes of the current study, both ACOD and PCOD changed, along with age, axial length and corneal diameter. Although on the whole, the changes of AOCD and PCOD were minor and not statistically significant, the range of changes was very wide. Serious changes in COD increase the difficulty of secondary in-the-bag IOL implantation, and related factors need to be furtherly studied. In our study, ΔCD represents lateral eye growth while ΔAL represents axial growth. The positive correlation between ΔACOD and ΔCD indicated that the change of ACOD was influenced by lateral eye growth rather than axial growth, since no correlation was shown between ΔACOD and ΔAL. While ΔPCOD did not show similar positive correlation with ΔCD, it changed strongly in agreement with ΔACOD. Meanwhile, ΔACOD and ΔPCOD both showed negative correlations with the interval between two surgeries, indicating a constriction trend of COD after primary surgery.

The strength to contract the capsule was more likely due to the proliferation of lens epithelial cells (LECs). Anterior and posterior capsule opacification were the classical fibrotic disease exhibiting LECs hyperproliferation and outgrow after cataract surgery in response to surgical trauma, myofibroblast formation, matrix deposition and constriction, which were considered to be associated with wrinkling/constriction of the residual capsule (13, 14). This surgery-induced complication was commonly seen in infants after congenital cataract surgery. In this fibrotic progression, the inflammation response had long been considered to be the inducement (15).

In our study, the correlations between ΔACOD and the concentrations of FGF-2 (*r*^2^ = 0.216, *y* = −0.04*x + 0.52, *P* = 0.037) and TGF-β1 (*r*^2^ = 0.225, *y* = 0.001*×−0.18, *P* = 0.013) prompted the roles of FGF-2 and TGF-β playing in LECs proliferation and differentiation. It had been shown that FGF-2 plays an important role in the proliferation and differentiation of LECs (16, 17). Low concentrations of FGF-2 could maintain LEC proliferation while high concentrations could promote differentiation and lentoid formation *in vitro* (17). Meanwhile, in the human lens, LECs transdifferentiated and proliferated into myofibroblasts through an epithelial-mesenchymal transition induced by TGF-β1, and as a result, these myofibroblasts promoted lens capsule wrinkling (18–20). Studies mentioned above proved that FGF-2 and TGF-β played roles in the progression of LECs proliferation and differentiation.

PDGF-AA (*r*^2^ = 0.108, *y* = 1.56 × 10^−3^*x − 0.58, *P* = 0.016) and VEGF (*r*^2^ = 0.080, *y* = 1.33 × 10^−3^*x − 0.29, *P* = 0.033) were the other two cytokines that showed correlations with ΔACOD, which indicated the participation of them in the change of residual capsule. Previously, Wu et al. found that the preoperative concentration of PDGF-AA exhibited positive correlations with the PCO scores at 1 month and 3 months after pediatric cataract surgery (*r* = 0.497, *P* = 0.030; *r* = 0.647, *P* = 0.009) (21). As for VEGF, which was considered strongly implicated in neovascularization, although there was no published study describing its association with the capsule, its existence in some nonvascular tissues, such as the cornea, was confirmed by Gogat et al. (22). To date, the concrete mechanism of the two cytokines in the change of residual capsule has been unknown, but our findings might provide new scope for further basic research.

The main strengths of our study are as follows. First, this study investigated the postoperative capsular outcome from the perspective of COD, and furtherly came up with a hypothesis that the change of ACOD after primary surgery was influenced by the lateral eye growth and postoperative inflammation. After primary surgery, the strength of the lateral eye growth gradually decreased as the patients passing the developmental phase after birth, but the strength to contract the capsule existed persistently. As a result, ACOD showed contraction trend after primary surgery. Second, more patients with congenital cataract were included in this study compared to those in previous studies. The limitations of our study must be mentioned. First, only 100 μl of aqueous humor was collected from a congenital cataract eye per surgery for security, which limited the number of cytokines that could be detected. Second, the results only presented the change in COD in interval between primary and secondary surgery, longer follow-up was needed.

In conclusion, the results showed that the COD remained changing after primary congenital cataract removal. The correlations between ΔACOD and ΔCD, the interval between two surgeries, as well as the correlations between ΔACOD and the concentrations of FGF-2, PDGF-AA, and TGF-β1, prompted us to first put forward the hypothesis that the change in ACOD was influenced by lateral eye growth and LECs proliferation regulated by cytokines. However, it is hard to summarize the changing rule of COD simply according to the result of this study, and thus further research is needed.

## Data Availability

The raw data supporting the conclusions of this article will be made available by the authors, without undue reservation.

## References

[1] Foster A Gilbert C Rahi J. Epidemiology of cataract in childhood: a global perspective. J Cataract Refract Surg. (1997) 23(Suppl 1):601–4. 10.1016/S0886-3350(97)80040-5 9278811

[2] Sheeladevi S Lawrenson JG Fielder AR Suttle CM. Global prevalence of childhood cataract: a systematic review. Eye (London, England). (2016) 30:1160–9. 10.1038/eye.2016.156 27518543 PMC5023808

[3] Kumar P Lambert SR. Evaluating the evidence for and against the use of IOLs in infants and young children. Expert Rev Med Devices. (2016) 13:381–9. 10.1586/17434440.2016.1153967 26878234 PMC4860524

[4] Solebo AL Cumberland P Rahi JS. 5-year Outcomes after primary intraocular lens implantation in children aged 2 years or younger with congenital or infantile cataract: findings from the IoLunder2 prospective inception cohort study. Lancet Child Adolesc Health. (2018) 2:863–71. 10.1016/S2352-4642(18)30317-1 30389448

[5] Chang DF Masket S Miller KM Braga-Mele R Little BC Mamalis N Complications of sulcus placement of single-piece acrylic intraocular lenses: recommendations for backup IOL implantation following posterior capsule rupture. J Cataract Refract Surg. (2009) 35:1445–58. 10.1016/j.jcrs.2009.04.027 19631134

[6] Mehta R Aref AA. Intraocular Lens implantation in the ciliary sulcus: challenges and risks. Clin Ophthalmol. (2019) 13:2317–23. 10.2147/OPTH.S205148 31819356 PMC6885568

[7] Trivedi RH Wilson ME Jr. Facciani J. Secondary intraocular lens implantation for pediatric aphakia. J AAPOS. (2005) 9:346–52. 10.1016/j.jaapos.2005.02.010 16102485

[8] Wilson ME Jr. Englert JA Greenwald MJ. In-the-bag secondary intraocular lens implantation in children. J AAPOS. (1999) 3:350–5. 10.1016/s1091-8531(99)70044-3 10613579

[9] Lin H Tan X Lin Z Chen J Luo L Wu X Capsular outcomes differ with capsulorhexis sizes after pediatric cataract surgery: a randomized controlled trial. Sci Rep. (2015) 5:16227. 10.1038/srep16227 26537991 PMC4633668

[10] Petric I Lacmanović Loncar V. Surgical technique and postoperative complications in pediatric cataract surgery: retrospective analysis of 21 cases. Croat Med J. (2004) 45:287–91. PMID: . 15185419

[11] Lambert SR Aakalu VK Hutchinson AK Pineles SL Galvin JA Heidary G Intraocular lens implantation during early childhood: a report by the American academy of ophthalmology. Ophthalmology. (2019) 126:1454–61. 10.1016/j.ophtha.2019.05.009 31230794

[12] Tan X Lin H Lin Z Chen J Tang X Luo L Capsular outcomes after pediatric cataract surgery without intraocular Lens implantation: qualitative classification and quantitative measurement. Medicine (Baltimore). (2016) 95:e2993. 10.1097/MD.0000000000002993 26962807 PMC4998888

[13] Bertelmann E Kojetinsky C. Posterior capsule opacification and anterior capsule opacification. Curr Opin Ophthalmol. (2001) 12:35–40. 10.1097/00055735-200102000-00007 11150079

[14] Wormstone IM Wang L Liu CS. Posterior capsule opacification. Exp Eye Res. (2009) 88:257–69. 10.1016/j.exer.2008.10.016 19013456

[15] Eldred JA Dawes LJ Wormstone IM. The lens as a model for fibrotic disease. Philos Trans R Soc Lond B Biol Sci. (2011) 366:1301–19. 10.1098/rstb.2010.0341 21402588 PMC3061111

[16] Lovicu FJ McAvoy JW. Structural analysis of lens epithelial explants induced to differentiate into fibres by fibroblast growth factor (FGF). Exp Eye Res. (1989) 49:479–94. 10.1016/0014-4835(89)90056-0 2792239

[17] Wang D Wang E Liu K Xia CH Li S Gong X. Roles of TGFβ and FGF signals during growth and differentiation of mouse lens epithelial cell in vitro. Sci Rep. (2017) 7:7274. 10.1038/s41598-017-07619-5 28779082 PMC5544739

[18] Novotny GE Pau H. Myofibroblast-like cells in human anterior capsular cataract. Virchows Arch A Pathol Anat Histopathol. (1984) 404:393–401. 10.1007/BF00695223 6437072

[19] Hales AM Chamberlain CG McAvoy JW. Cataract induction in lenses cultured with transforming growth factor-beta. Invest Ophthalmol Visual Sci. (1995) 36:1709–13. PMID: . 7601651

[20] Lovicu FJ Schulz MW Hales AM Vincent LN Overbeek PA Chamberlain CG TGFbeta induces morphological and molecular changes similar to human anterior subcapsular cataract. Br J Ophthalmol. (2002) 86:220–6. 10.1136/bjo.86.2.220 11815351 PMC1771017

[21] Wu X Liu Z Wang D Lin D Long E Lin Z Preoperative profile of inflammatory factors in aqueous humor correlates with postoperative inflammatory response in patients with congenital cataract. Mol Vision. (2018) 24:414–24. PMID: . 29930475 PMC5993531

[22] Gogat K Le Gat L Van Den Berghe L Marchant D Kobetz A Gadin S VEGF And KDR gene expression during human embryonic and fetal eye development. Invest Ophthalmol Visual Sci. (2004) 45:7–14. 10.1167/iovs.02-1096 14691147

